# Parkinson’s LRRK2-G2019S risk gene mutation drives sex-specific behavioral and cellular adaptations to chronic variable stress

**DOI:** 10.3389/fnbeh.2024.1445184

**Published:** 2024-09-12

**Authors:** Christopher A. Guevara, Kumayl Alloo, Swati Gupta, Romario Thomas, Pamela del Valle, Alexandra R. Magee, Deanna L. Benson, George W. Huntley

**Affiliations:** ^1^Nash Family Department of Neuroscience, New York, NY, United States; ^2^Friedman Brain Institute, New York, NY, United States; ^3^Graduate School of Biomedical Sciences, Icahn School of Medicine at Mount Sinai, New York, NY, United States; ^4^Department of Biological Sciences, Columbia University, New York, NY, United States

**Keywords:** anxiety, depression, mice, open-field, novelty-suppressed feeding, social interaction, cFos, activity-mapping

## Abstract

Anxiety is a psychiatric non-motor symptom of Parkinson’s that can appear in the prodromal period, prior to significant loss of midbrain dopamine neurons and motor symptoms. Parkinson’s-related anxiety affects females more than males, despite the greater prevalence of Parkinson’s in males. How stress, anxiety and Parkinson’s are related and the basis for a sex-specific impact of stress in Parkinson’s are not clear. We addressed this using young adult male and female mice carrying a G2019S knockin mutation of leucine-rich repeat kinase 2 (*Lrrk2*^G2019S^) and *Lrrk2*^WT^ control mice. In humans, *LRRK2*^G2019S^ significantly elevates the risk of late-onset Parkinson’s. To assess within-sex differences between *Lrrk2*^G2019S^ and control mice in stress-induced anxiety-like behaviors in young adulthood, we used a within-subject design whereby *Lrrk2*^G2019S^ and *Lrrk2*^WT^ control mice underwent tests of anxiety-like behaviors before (baseline) and following a 28 day (d) variable stress paradigm. There were no differences in behavioral measures between genotypes in males or females at baseline, indicating that the mutation alone does not produce anxiety-like responses. Following chronic stress, male *Lrrk2*^G2019S^ mice were affected similarly to male wildtypes except for novelty-suppressed feeding, where stress had no impact on *Lrrk2*^G2019S^ mice while significantly increasing latency to feed in *Lrrk2*^WT^ control mice. Female *Lrrk2*^G2019S^ mice were impacted by chronic stress similarly to wildtype females across all behavioral measures. Subsequent post-stress analyses compared cFos immunolabeling-based cellular activity patterns across several stress-relevant brain regions. The density of cFos-activated neurons across brain regions in both male and female *Lrrk2*^G2019S^ mice was generally lower compared to stressed *Lrrk2*^WT^ mice, except for the nucleus accumbens of male *Lrrk2*^G2019S^ mice, where cFos-labeled cell density was significantly higher than all other groups. Together, these data suggest that the *Lrrk2*^G2019S^ mutation differentially impacts anxiety-like behavioral responses to chronic stress in males and females that may reflect sex-specific adaptations observed in circuit activation patterns in some, but not all stress-related brain regions.

## 1 Introduction

Anxiety is a prevalent psychiatric non-motor symptom of genetic and idiopathic forms of Parkinson’s that can appear in the prodromal stage, before significant dopamine neuron degeneration (Shiba et al., 2000; Broen et al., 2016). Chronic behavioral stress has emerged as an important risk factor for psychiatric symptoms and Parkinson’s (Hemmerle et al., 2012). How stress, psychiatric symptoms and Parkinson’s are related mechanistically is unknown. Mice carrying gene mutations associated with an increased risk for Parkinson’s provide an opportunity to examine the intersection between genetic and experience-dependent risks.

The G2019S mutation in *LRRK2* (encoding leucine-rich repeat kinase 2) is among the most common genetic risk mutations for familial and sporadic Parkinson’s (Kluss et al., 2019). *LRRK2*^G2019S^ produces late-onset Parkinson’s that is clinically similar to idiopathic forms, including prodromal anxiety and depression in non-manifesting mutation carriers (Shanker et al., 2011; DeBroff et al., 2023). Genetic, pathological, and preclinical studies suggest that dysregulated LRRK2 may be part of a common pathological mechanism (Kluss et al., 2019; Rocha et al., 2022; Peter and Strober, 2023). In brain, LRRK2 protein is enriched in areas that are susceptible or can adapt negatively to stressful behavioral experiences, including medial prefrontal cortex (mPFC), amygdala, hippocampus and striatum (Simón-Sánchez et al., 2006; Giesert et al., 2013), suggesting that LRRK2 is well-positioned to modify responses to stress.

Previous work has shown that male *Lrrk2*^G2019S^ knockin mice display altered social interaction and hedonic responses following periods of social defeat stress that are distinct from wildtype mice (Matikainen-Ankney et al., 2018; Guevara et al., 2020). Social defeat stress is an intense stressor that has most readily been applied to males. This leads us to ask whether such synergy between social stress and *Lrrk2*^G019S^ can be generalized to different durations and forms of stress. We also address the effects of stress on female *Lrrk2*^G2019S^ mice. To answer these questions, we mounted a fully powered comparison of anxiety-like behaviors and examined brain regions activated in males and females following extended exposure to a chronic variable stress protocol.

## 2 Methods

### 2.1 Mice

*Lrrk2*^G2019S^ knockin mice (RRID:MGI:6273311) were generated by Eli Lilly and characterized previously (Matikainen-Ankney et al., 2016, 2018; Guevara et al., 2020; Hussein et al., 2022). The knockin mice were congenic on C57Bl/6NTac background, bred as homozygotes, and backcrossed to wildtype C57Bl/6NTac every fifth generation to prevent genetic drift. Age-, sex- and strain-matched wildtype mice (*Lrrk2*^WT^) were used as controls. All mice were bred in Mount Sinai’s vivarium under identical conditions. The mice were group housed and maintained on a 12 h light/dark cycle with free access to food and water unless otherwise specified. Genotypes of all animals were confirmed through PCR and sequencing. Male and female heterozygous *Lrrk2*^G2019S^ mice and age- and strain-matched wildtype mice were utilized for all experiments. Heterozygotes were generated by crossing *Lrrk2*^G2019S/G2019S^ mice with *Lrrk2*^WT/WT^ mice. *LRRK2*^G2019S^ causes autosomal dominant Parkinson’s in human carriers, with one or two copies of the mutation producing a similar disease course (Lesage et al., 2007; Healy et al., 2008; Yahalom et al., 2014). In line with this, previous studies have shown similar behavioral and electrophysiological outcomes between hetero- and homozygotes (Matikainen-Ankney et al., 2016; Guevara et al., 2020). Mice were aged postnatal day 56–60 at the start of the behavioral experiments. In the females, no attempt was made to document the stage of the estrous cycle in relation to behavioral testing. Recent studies suggest the impact of the estrous cycle on behavioral performance in tests of anxiety, including those used in this study, is negligible (Chari et al., 2020; Johnson et al., 2021; Levy et al., 2023; Li et al., 2023). On the other hand, another recent study showed that VTA dopamine neuron physiology is sensitive to phases of the estrous cycle, and that cycling sex steroids impact the stress-sensitivity of dopamine-related reward behaviors (Shanley et al., 2023). All experiments were approved by Mount Sinai’s Institutional Animal Care and Use Committee and conformed to National Institutes of Health guidelines.

### 2.2 Experimental design of behavioral testing

**Figure 1:**
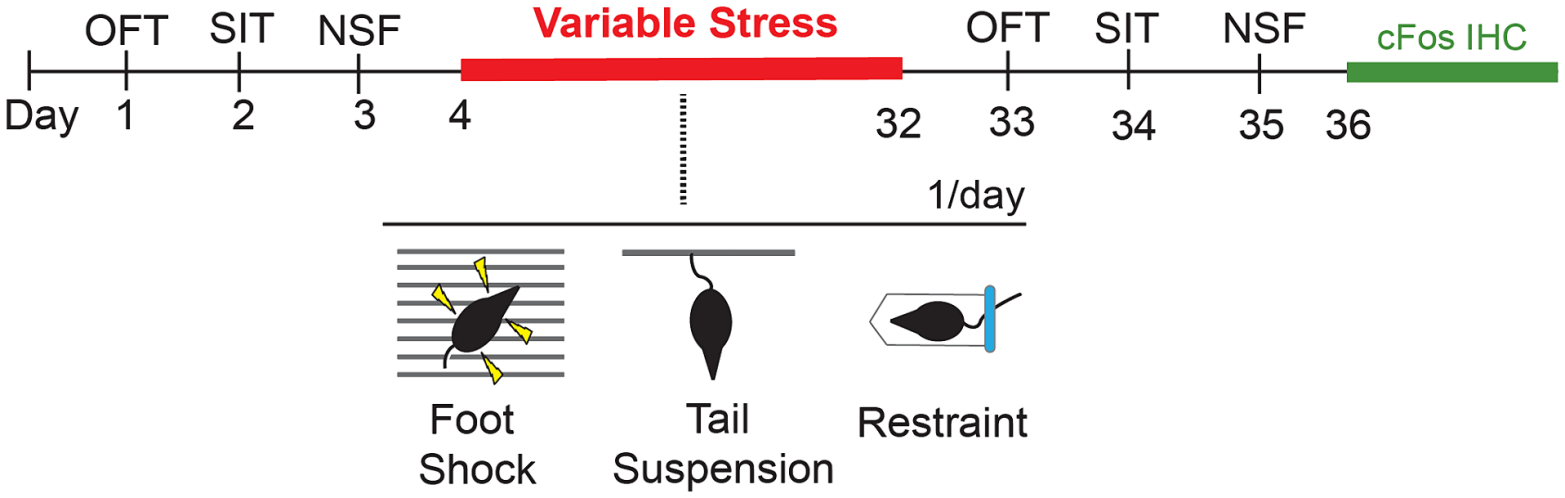
Schematic showing timeline of behavioral tests prior to (baseline) and following a 28d variable stress paradigm, with subsequent cFos immunohistochemistry (IHC). OFT, Open Field Test; SIT, Social Interaction Test; NSF, Novelty Suppressed Feeding.

We used a within-subject design to evaluate the effects of 28d of variable stress on different measures of anxiety-like behaviors. To establish a pre-stress baseline of responses, behaviorally naïve *Lrrk2*^G2019S^ and *Lrrk2*^WT^ mice were tested on a battery of standard tests on days 1–3. Twenty-four hours later, mice were subjected to 28d of variable stress. Twenty-four hours after the last day of the stress paradigm, mice were re-tested on the same daily pre-stress behavioral battery to establish post-stress effects for comparison (Figure 1). The pre- and post-stress behavioral assays comprised an open field test, a social interaction test with a same-sex conspecific, and a novelty-suppressed feeding test. One test was conducted per day and animals were allowed to habituate to the testing room for 1 h prior to testing. Equipment was cleaned with 70% ethanol in between testing sessions. Mice were group-housed throughout the entire experiment (Westenbroek et al., 2005; Manouze et al., 2019).

### 2.3 Open field test (OFT)

Mice were placed in an open arena (40 × 40 × 30 cm) under white light and allowed to explore for 5 min (Cunningham et al., 2021). We designated a 20.3 × 20.3 cm square centrally located within the arena as the “center” with the rest of the arena designated as the “periphery.” Fusion Software (v5.0) (Omnitech Electronics) was used to quantify time spent in the center and periphery of the open arena as an indicator of anxiety (Badimon et al., 2020), and to monitor total distance traveled.

### 2.4 Social interaction test (SIT)

Social interaction (SI) was assessed in two 150 s phases, separated by a duration of 30 s, either in the absence or presence of a novel conspecific social target under red lights (Muir et al., 2020; Tsyglakova et al., 2021). SI behavior was calculated as an SI Ratio = t_present_/t_absent_, where t_present_ and t_absent_ represent the times spent in the interaction zone in the presence and absence of the novel social target. Time and location in the arena were recorded by video tracking software (Ethovision 10.0, Noldus).

### 2.5 Novelty suppressed feeding (NSF)

NSF tests were conducted as described (Freire-Cobo and Wang, 2020; Johnson et al., 2021). Briefly, mice were food-deprived the night before the test. On the day of testing, mice were placed in an arena containing a single food pellet in the center. The latency to take a bite from the piece of food was scored manually for a maximum of 10 min. The test was conducted under red light. All mice were weighed in the evening prior to food restriction and the next morning prior to the NSF test.

### 2.6 Variable stress paradigm

The variable stress paradigm was performed as previously described (Freire-Cobo and Wang, 2020; Johnson et al., 2021). Mice were subjected to one of three stressors in the following order for 1 h per day: 100 random foot shocks of 0.45 mA for 3 s each (day 1), tail suspension (day 2), and restraint by placing the animal in a ventilated 50-mL conical tube (day 3). Stressors were administered and repeated in this order over 28d. Mice were returned to their homecage and group-housed at the end of each stressor.

### 2.7 cFos activity mapping and immunohistochemistry

Twenty-four hours following the last behavioral assay, 4–5 mice of each sex and genotype were subjected to restraint stress for 1 h then returned to their homecage for 90 min followed by transcardial perfusion with 4% paraformaldehyde in phosphate buffered saline (PBS, pH7.3) (Figure 1). Following 6 h post-fixation, brains were sectioned coronally at 50 μM on a freezing sliding microtome (Leica). Immunocytochemical localization of cFos was performed on every sixth section (approximately every 300 μm). Free-floating sections were incubated in 2% bovine serum albumin (BSA) in 0.3% Tween-20 PBS for 1 h then incubated in rabbit anti-c-Fos antibody (1:500, Synaptic Systems, catalog # 226–003, RRID: AB_2231974) diluted in the same solution for 72 h at 4°C. Sections were washed, then incubated in goat anti-rabbit Alexa 647-conjugated antibodies (1:500, Abcam catalog #150076, RRID: AB_2782993) diluted in 2% BSA in 0.3% Tween-20 PBS for 1 h and then stained with 0.005% DAPI in PBS for 20 min. Finally, sections were slide-mounted and coverslipped with Mowiol.

### 2.8 Brain regions chosen for cFos activity-mapping

Stress is a significant risk factor for anxiety and depression in humans (Hemmerle et al., 2012), and a large number of brain regions have been implicated in anxiety- and depression-like behaviors (Russo and Nestler, 2013). We chose a subset of potential brain regions for cFos analysis that included mPFC (areas PL and IL in mice), dorsal striatum (dStr), nucleus accumbens (NAc), basolateral amygdala (BLA), dorsal hippocampal area CA1 (dHPC) and ventral hippocampal area CA1 (vHPC). This subset of areas met two criteria for analysis: they respond to stress and/or have been implicated in anxiety-like or depression-like behaviors in mice and humans (Furman et al., 2011; Gabbay et al., 2013; Kerestes et al., 2015; Cooper et al., 2017; Muir et al., 2020; Kim and Chung, 2021; Rainville et al., 2022; Giovanniello et al., 2023) and they exhibit significant LRRK2 expression (Taymans et al., 2006; Giesert et al., 2013; Huntley and Benson, 2020).

### 2.9 Data and image analysis

Single-plane, tiled images of entire cFos-immunolabeled brain sections were captured on a Leica DMi8 widefield microscope equipped with a 10X objective lens, a SpectraX fluorescence illuminator, and a Leica DFC9000GT monochrome camera in the Microscopy and Advanced Bioimaging Core at Mount Sinai. Anatomical regions were identified using Nissl staining as a guide, and a region of interest (ROI) of the same size was generated in each section across mice. In layered structures (PL, IL, hippocampus), ROIs included all layers.

Density of cFos-labeled cells was quantified using the “Cell Counter” plug-in on ImageJ/Fiji. Thresholding limits were determined using the “Moments” setting on ImageJ/Fiji. cFos density was calculated as # cFos-labeled cells/area of ROI (μm^2^).

### 2.10 Statistical analyses

The numbers of animals used for behavioral and cFos mapping experiments are given in the relevant figure legends. Statistical comparisons of normally distributed data between two groups were conducted using two-tailed Student’s t-tests. To compare parametric data from multiple groups, we used two-way ANOVA, but for within-subject comparisons of parametric data from multiple groups, we used two-way repeated-measures ANOVA followed by Šidák’s *post-hoc* test for multiple comparisons. For nonparametric data between two groups, a Mann–Whitney U test was used. For all datasets, we tested for outliers using Grubb’s test, but no animal was excluded because no single animal was an outlier on all behavioral tests. Analyses were conducted using GraphPad Prism (v8.2.1), with *p* < 0.05 considered significant. Data are presented as means ± SEM.

## 3 Results

We subjected young adult male and female *Lrrk2*^WT^ and heterozygous *Lrrk2*^G2019S^ knockin mice to a variable stress (*VS*) paradigm for 28 days (28d-*VS*) to assess the impact of stress, sex and genetic risk for Parkinson’s on measures of anxiety-like behaviors in mice using a within-subject design to compare baseline (pre-stress) and post-stress responses (Figure 1).

### 3.1 Open field test (OFT)

**Figure 2:**
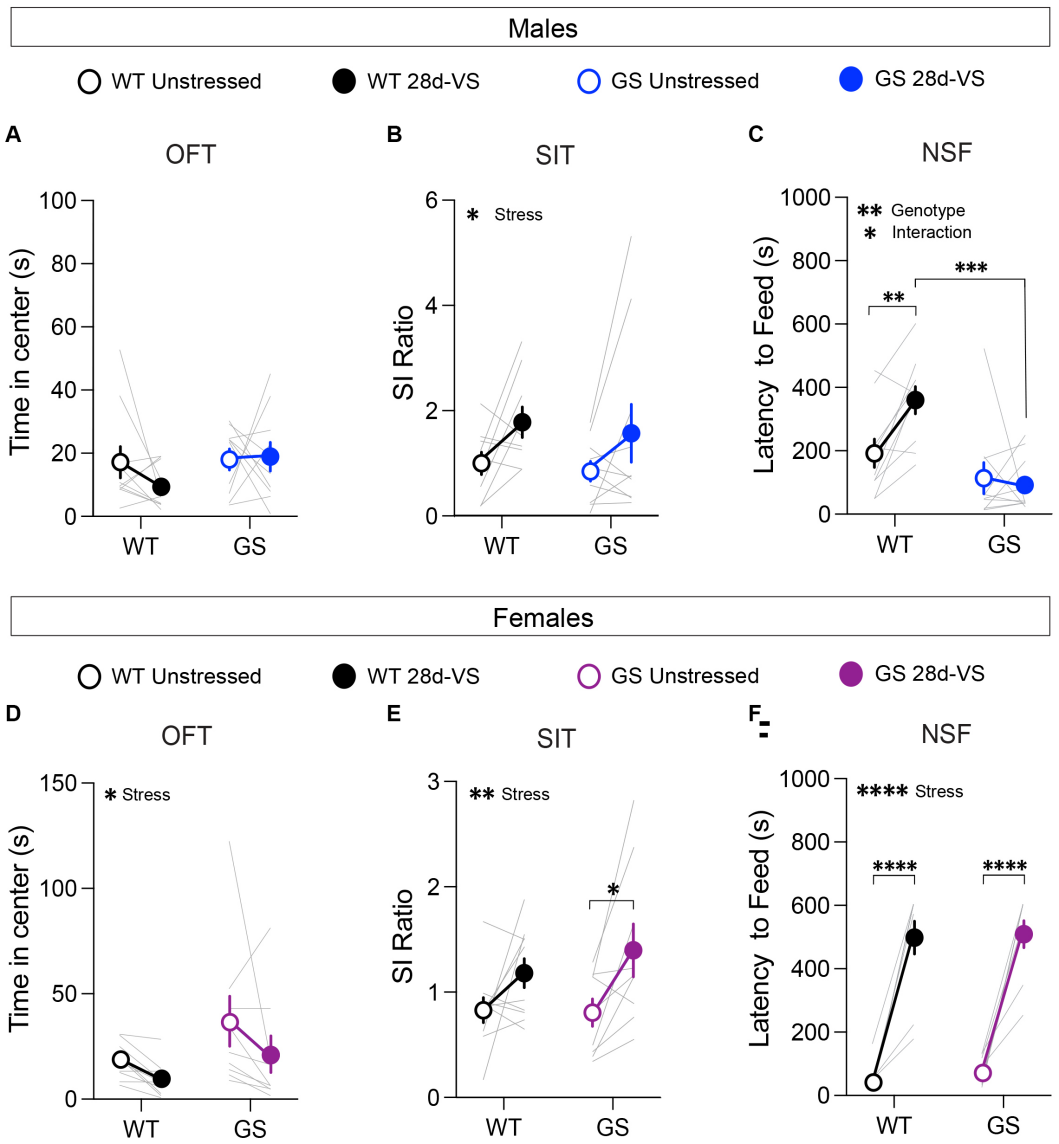
Effects of 28d-*VS* (variable stress) on measures of anxiety-like behavior, social interaction and hyponeophagia in males (top row) and females (bottom row). **(A)** Male *Lrrk2*^WT^ (WT) control and *Lrrk2*^G2019S^ (GS) mice showed no changes to time in center following 28d-*VS* in comparison with their baseline measures (*p* = 0.4293, F_1,18_ = 0.6539 for main effect of stress). **(B)** WT and GS males both exhibited an increased social interaction (SI) ratio following 28d-*VS* in comparison with baseline SI ratios (*p* = 0.0201, F_1,17_ = 6.572 for main effect of stress). **(C)** In an NSF test, GS males were unaffected by 28d-*VS* compared to their baseline latencies. In contrast, WT males showed a significantly increased latency to feed (*p* = 0.0099), which was significantly longer than post-stress latencies of GS males (*p* = 0.0001) (*p* = 0.0199, F_1,18_ = 0.2994 for interaction effect; *p* = 0.0014, F_1,18_ = 14.21 for main effect of genotype). **(D)** Both WT and GS females show a decreased time in center following 28d-*VS* (*p* = 0.046, F_1,17_ = 4.635 for main effect of stress). **(E)** WT and GS females both showed an increased SI ratio following 28d-*VS* (*p* = 0.0036, F_1,17_ = 11.42 for main effect of stress); with GS females showing a significantly increased SI ratio compared to their baseline measures (*p* = 0.019). **(F)** Both WT and GS female mice exhibited a similarly increased latency to feed following 28d-*VS* (*p* = <0.0001) (*p* = <0.0001, F_1,17_ = 143.7 for main effect of stress). *n* = 10 WT male mice, 10 WT female mice, 13 GS male mice, and 9 GS female mice. Statistical tests used: 2-way repeated measures ANOVA with *post hoc* Šidák test for multiple comparisons. Data presented as mean ± s.e.m. *p < 0.05; **p < 0.01; ***p < 0.001, ****p < 0.001.

In an OFT, reduced time spent in the center of an open arena is interpreted as increased anxiety-like behavior (Simon et al., 1994; Harro, 2018). At baseline, we found no significant genotype differences among the male and female cohorts in the time spent in the center (Figures 2A,D) or in total distance traveled, indicating that the mutation alone does not significantly affect anxiety-like behavior or locomotor activity in this test in males or females, as expected (Yue et al., 2015). After 28d-*VS*, male *Lrrk2*^WT^ and *Lrrk2*^G2019S^ mice exhibited no significant stress-related changes in the time spent in the center (Figure 2A). However, in contrast to males, both *Lrrk2*^WT^ and *Lrrk2*^G2019S^ female mice showed a main effect of stress by a significantly decreased time in center following 28d-*VS* (Figure 2D). There were no significant effects of stress on locomotor activity in males or females (males: main effect of stress, F_1,18_ = 1.267, *p* = 0.054, main effect of genotype, F_1,18_ = 0.1268, *p* = 0.7259; females: main effect of stress, F_1,17_ = 1.807, *p* = 0.1966, main effect of genotype, F_1,17_ = 1.063, *p* = 0.3169).

### 3.2 Social interaction test (SIT)

Mice were tested for social interaction (SI) behavior with an age- and sex-matched conspecific because social avoidance is a measure of anxiety- and depression-like states (Golden et al., 2011). There were no baseline genotype differences in SI ratios in males or females (Figures 2B,E), as expected (Matikainen-Ankney et al., 2018; Guevara et al., 2020). However, there was a main effect of 28d-*VS* on SI behavior in both genotypes and sexes (Figures 2B,E). In males and females, we found that 28d-*VS* significantly increased social interaction in *Lrrk2*^WT^ and *Lrrk2*^G2019S^ mice. *Post hoc* analysis of the female cohort showed that female *Lrrk2*^G2019S^ mice had a significantly greater increase in SI ratios following 28d-*VS* compared to their baselines (Figure 2E).

### 3.3 Novelty suppressed feeding (NSF)

Mice were food-restricted overnight and subsequently placed in a novel arena. Their latency to feed on a new food pellet was measured. Increased latency to feed is interpreted as a measure of heightened anxiety-like behavior because this measure is reduced by anxiolytic drugs (Bodnoff et al., 1989). Under baseline conditions, there were no genotype differences in males or females in response latencies to feed (Figures 2C,F). In males, *post hoc* analyses showed that following 28d-*VS*, *Lrrk2*^WT^ mice exhibited significantly increased latencies to feed compared to their pre-stress baseline responses (Figure 2C). In contrast, male *Lrrk2*^G2019S^ showed no significant differences between unstressed and post-stress latencies to feed (Figure 2C), suggesting a lack of anxiety-like effect in this test driven by 28d-*VS* compared to a significant elevation in anxiety-like behavior of *Lrrk2*^WT^ mice. We verified that in the male mice, there were no genotype differences in the percent body weight lost after overnight fasting, either under baseline or post-28d-*VS* conditions (repeated-measures two-way ANOVA, main effect of genotype, F_1,18_ = 0.2795, *p* = 0.6035), ruling out potential differences between genotypes in weight lost as a factor accounting for different post-stress effects on latency to feed.

In female mice, there was a significant effect of stress on latencies to feed that was evident in both genotypes, with *Lrrk2*^WT^ and *Lrrk2*^G2019S^ female mice exhibiting significantly increased latencies to feed following 28d-*VS* compared to their unstressed baseline latencies (Figure 2F). Comparison of overnight fasting-induced body weight loss showed that at baseline, female *Lrrk2*^G2019S^ mice lost a significantly greater percentage of body weight compared to baseline measured in female wildtype mice (two-way repeated-measures two-way ANOVA, main effect of genotype, F_1,17_ = 9.262, *p* = 0.0073), but there were no significant differences between genotypes in body weight lost following 28d-VS. As the latencies to feed were similar between genotypes under both baseline and post-stress conditions, the significance of a greater percentage of body weight lost at baseline in the *Lrrk2*^G2019S^ females is not clear. In sum, following stress, latency to feed was increased significantly in both male and female wildtype mice and in female *Lrrk2*^G2019S^ mice. Male *Lrrk2*^G2019S^ mice were unaffected.

**Figure 3:**
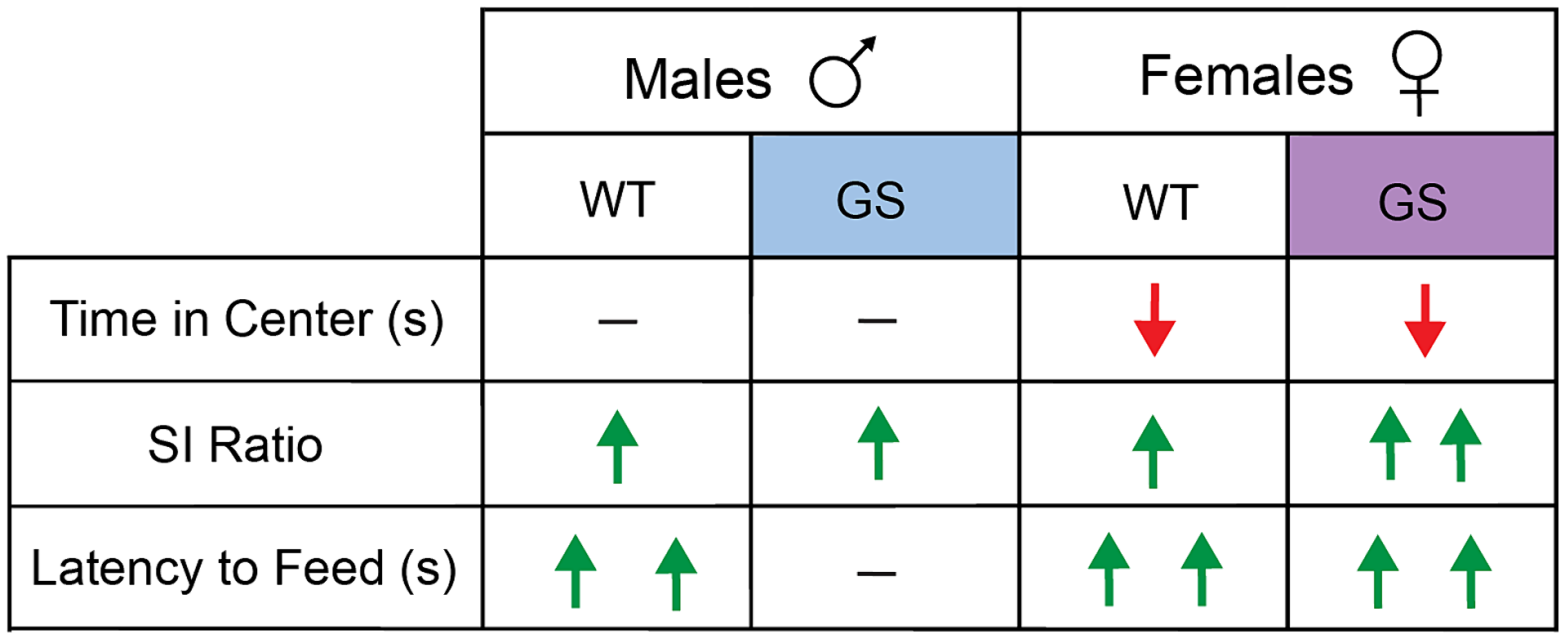
Summary of effects of 28d-*VS* on WT and GS male and female mice across all behavioral measures. Directionality of change in behavioral performance following 28d-*VS* is indicated by color and direction of arrows. Red downward arrow = decreased measure of behavior performance following 28d-*VS* compared to baseline measures; green upward arrow = increased measure of behavioral performance following 28d-*VS* compared to baseline measures; horizontal line = no change in measure of behavioral performance following 28d-VS. More than one arrow = significance in *post-hoc* comparison.

A summary of behavioral results, following 28d-*VS* in males and females, is shown in Figure 3.

### 3.4 Cellular activity mapping by cFos immunolabeling following 28d-*VS*

**Figure 4:**
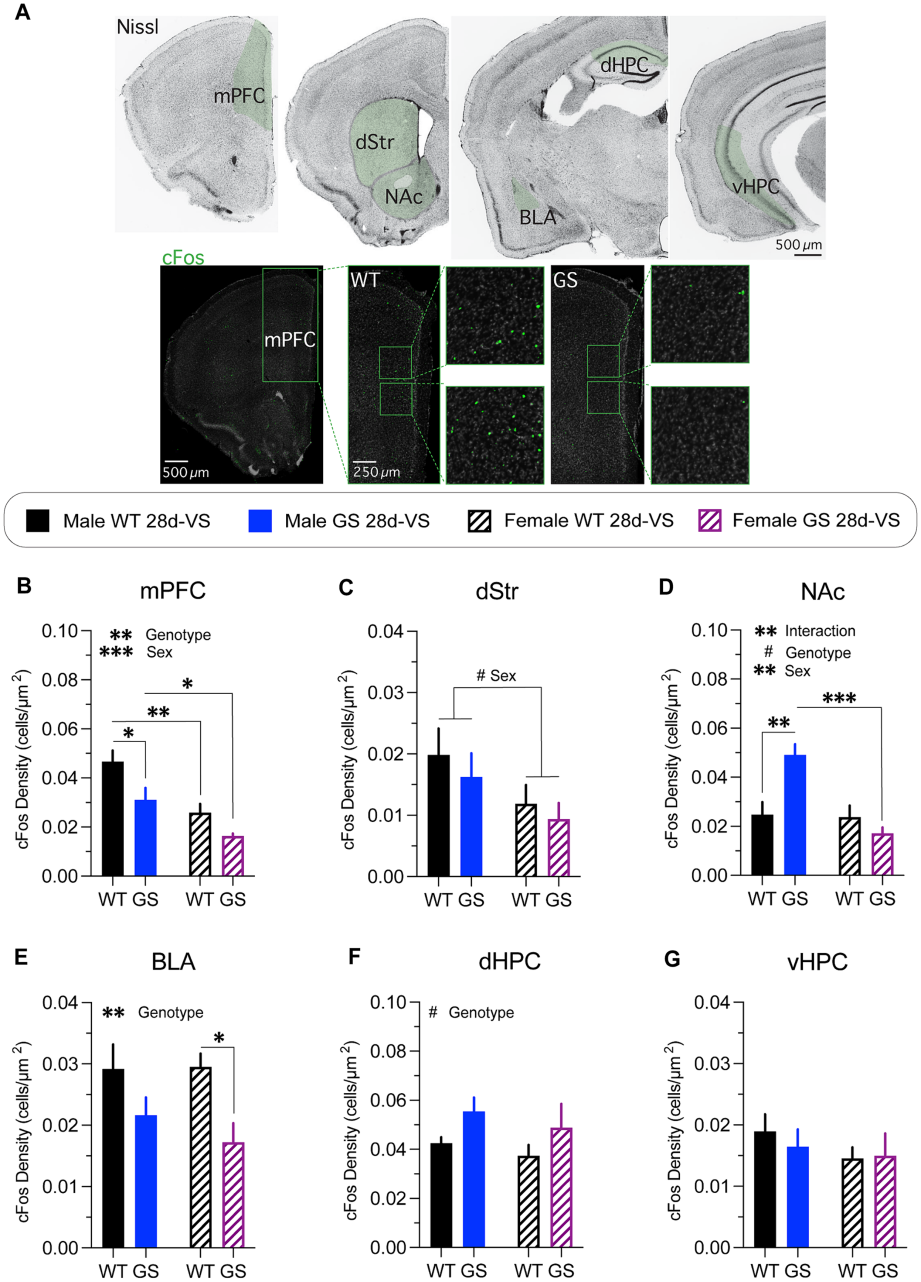
cFos immunolabeling-based cellular activity patterns in male and female mice following stress. cFos immunofluorescent labeling of perfused brain sections was used as an activity marker in stress-relevant brain regions following stress-experience. **(A)** A Nissl-stained series of frontal sections (top row) through the brain of a wildtype mouse post-stress with regions in green shading highlighting those analyzed for cFos immunolabeling. Representative confocal microscope images through mPFC of a male WT mouse (bottom row, left) or of a male GS mouse (bottom row, right) showing examples of cFos immunofluorescently-labeled cells (boxed regions show higher-magnification images). mPFC, medial prefrontal cortex; dStr, dorsal striatum; NAc, nucleus accumbens; BLA, basolateral amygdala; dHPC, dorsal hippocampus; vHPC, ventral hippocampus. Following stress, mice exhibited differential genotype and sex differences in most areas examined. **(B)** Analysis of cFos-labeled cell density in mPFC post-stress (*p* = 0.0085, F_1,14_ = 9.345 for genotype effect; *p* = 0.0006, F_1,14_ = 19.06 for main effect of sex); with GS males (*p* = 0.0258) and WT females (*p* = 0.0057) showing decreased density of cFos-labeled cells compared to WT males, and GS females showing decreased density of cFos-labeled cells compared to GS males (*p* = 0.0442). **(C)** Analysis of cFos-labeled cell density in dStr post-stress (*p* = 0.0657, F_1,14_ = 3.985 for main effect of sex). **(D)** Analysis of cFos-labeled cell density in NAc post-stress (*p* = 0.0037, F_1,14_ = 12.05 for interaction effect; *p* = 0.0672, F_1,14_ = 3.936 for genotype effect; *p* = 0.0024, F_1,14_ = 13.68 for main effect of sex); with GS males (*p* = 0.0022) showing increase cFos-labeled cell density compared to WT males, and GS females showing decreased density of cFos-labeled cells compared to GS males (*p* = 0.0003). **(E)** Analysis of cFos-labeled cell density in BLA post-stress (*p* = 0.0088, F_1,14_ = 9.261 for genotype effect); with GS females showing a significantly decreased density of cFos-labeled cells compared to WT females (*p* = 0.0474). **(F)** Analysis of cFos-labeled cell density in dHPC post-stress (*p* = 0.0667, F_1,13_ = 4.005 for main effect of genotype). **(G)** Analysis of cFos-labeled cell density in vHPC post-stress mice (*p* = 0.62, *F*_(1,14)_ = 0.25 for interaction effect; *p* = 0.72, F_(1,14)_ = 0.13 for genotype effect; *p* = 0.33, F_(1,14)_ = 0.10 for sex effect). For all experiments, n = average of 2 brain slices per mouse / 4–5 mice per group. Statistical tests used: 2-way ANOVA with *post hoc* Šidák test for multiple comparisons. Data presented as mean ± s.e.m. ^#^*p* = 0.05–0.07, *p < 0.05; **p < 0.01; ***p < 0.001.

To assay sex and genetic differences in regional activation patterns of the stress-network following stress-experience, we used immunolabeling for the immediate early gene cFos as a neural activity marker (Morgan and Curran, 1989) (Figure 4A). We analyzed a subset of brain regions implicated in anxiety-like behaviors and that exhibit high levels of LRRK2 expression (see Materials and Methods).

Both male and female *Lrrk2*^G2019S^ mice had a significantly decreased density of cFos immunofluorescent cells in mPFC following stress compared to their respective stressed *Lrrk2*^WT^ controls (Figure 4B), with combined males having a significantly higher cFos-labeled cell density overall in comparison with combined females (Figure 4B). In dStr (Figure 4C), there was a trending main effect of sex with combined males having higher densities of cFos-labeled cells compared to combined females. In NAc (Figure 4D), there was a significant increase in the density of cFos immunolabeled cells in male *Lrrk2*^G2019S^ mice compared to male *Lrrk2*^WT^ mice, but no significant differences in cFos-labeled cell density between *Lrrk2*^G2019S^ and *Lrrk2*^WT^ female mice (Figure 4D). Together, these data support a main effect of genotype, sex and their interaction. In BLA (Figure 4E), there was a main effect of genotype, with lower cFos-labeled cell densities in *Lrrk2*^G2019S^ mice compared to *Lrrk2*^WT^ mice and significantly lower cFos-labeled cell density in stressed *Lrrk2*^G2019S^ female mice compared to stressed female *Lrrk2*^WT^ mice. In dHPC (Figure 4F), there was a trending main effect of genotype with higher densities of cFos-labeled cells in *Lrrk2*^G2019S^ mice compared to *Lrrk2*^WT^ controls, while in vHPC, there were no genotype or sex-related differences in cFos-labeled cells following stress (Figure 4G). Together, these data suggest that the recruitment of neural networks across some stress-responsive brain regions differs between male and female mice. c-Fos-based activation levels appeared to be generally higher in male than in female mice. Most striking was the selectively increased activation of NAc in male *Lrrk2*^G2019S^ mice because, outside of this, the mutation appeared to mostly dampen activation.

## 4 Discussion

The purpose of this study was to better understand how the Parkinson’s *Lrrk2*^G2019S^ risk mutation impacted stress-induced anxiety-like behaviors in young adulthood in males and females. We subjected male and female *Lrrk2*^G2019S^ heterozygous knockin and *Lrrk2*^WT^ control mice to a 28d chronic variable stress paradigm to assess within-sex differences between *Lrrk2*^G2019S^ mice and *Lrrk2*^WT^ controls. Using several tests of anxiety-like behaviors, we found that in males, chronic variable stress affected behavioral performance of *Lrrk2*^WT^ and *Lrrk2*^G2019S^ mice similarly with the exception of an NSF test, where stress had no impact on *Lrrk2*^G2019S^ mice while significantly affecting wildtypes. Female *Lrrk2*^G2019S^ mice were impacted by chronic stress similarly to *Lrrk2*^WT^ control females across all behavioral measures. Subsequent post-stress analyses compared cFos immunolabeling-based cellular activity patterns across several stress-relevant brain regions. We found that in male *Lrrk2*^G2019S^ mice, post-stress patterns of cFos-labeled cells in several areas, including mPFC and the NAc, differed significantly from those found in either stressed *Lrrk2*^WT^ males or stressed *Lrrk2*^G2019S^ females. In contrast, stressed female *Lrrk2*^G2019S^ mice showed cFos-labeling patterns that were mostly similar to stressed female wildtypes with the exception of the BLA, which had significantly fewer cFos-labeled neurons in stressed female *Lrrk2*^G2019S^ mice compared to stressed female *Lrrk2*^WT^ mice. Together, these data suggest that the *Lrrk2*^G2019S^ mutation differentially impacts anxiety-like behavioral responses to chronic stress in male and female mutation carriers. This may reflect sex-specific adaptations in circuit activation patterns in many, but not all, stress-related brain regions. More broadly, our findings suggest a potential basis for differences in the onset and/or severity of psychiatric symptoms in male and female Parkinson’s patients.

We used a within-subject longitudinal behavioral testing design because it greatly increases statistical power over a between-subjects design (Partridge et al., 2023). Moreover, a within-subject behavioral testing design has been used in several previous studies of the effects of stress on anxiety-like measures in mice, including some of the measures used in the present study (Muir et al., 2020; Morel et al., 2022). It has been shown that some behavioral tests (e.g., elevated plus maze) are sensitive to repeated exposure (McIlwain et al., 2001) and inter-exposure interval (Paylor et al., 2006). While we cannot rule out the possibility of effects on post-stress behaviors following the initial baseline exposure, the possibility of significant repeat-exposure effects on post-stress behaviors is unlikely. First, the effects of 28d-*VS* on anxiety-like behaviors of our *Lrrk2*^WT^ control mice were similar to previous studies of wildtype mice that used a between-subject design. For example, we found a main effect of stress in the significantly longer latencies to feed in the NSF test in male and female *Lrrk2*^WT^ mice as reported previously with shorter (Bittar et al., 2021) or identical periods of exposure to similar stressors (Johnson et al., 2021). Similarly, social interaction time with a sex-matched, conspecific target increased in both male and female *Lrrk2*^WT^ mice following 28d-*VS* as reported previously (Zhao et al., 2020; LeClair et al., 2021; Li et al., 2023). Second, all mice were subjected to the identical protocol, so any effect of prior test exposure would be expected to affect all mice. Importantly, in the absence of stress, we found no within-sex differences between *Lrrk2*^G2019S^ knockin mice and their wildtype controls at baseline. This observation indicates that the mutation does not, in and of itself, drive aberrant anxiety-like, depression-like or anhedonia-like behaviors in either male or female *Lrrk2*^G2019S^ mice at the young adult ages examined, as shown previously (Yue et al., 2015; Volta et al., 2017; Matikainen-Ankney et al., 2018; Guevara et al., 2020). While there were no baseline behavioral effects of the mutation at young adult ages, it is possible that in the absence of stress, mutation-driven effects on anxiety- or depression-like behaviors would emerge with advancing age. In a study using BAC transgenic mice overexpressing human *LRRK2*^G2019S^, anxiety- and depression-like behaviors that were absent in young adulthood first appeared in both male and female mutant mice at about 1 year of age—in the absence of overt stress—and persisted at older ages (Lim et al., 2018). Moreover, such age-dependent effects on psychiatric-like behaviors in these transgenic mice were correlated with contemporaneous changes in hippocampal serotonin levels and altered serotonergic receptor expression (Lim et al., 2018). Setting aside significant differences in the genetics of the two mouse models, it may be that the knockin mice may gradually show an age-dependent effect on anxiety-like behaviors in the absence of stress. Future studies will be needed to examine this and to see if serotonergic innervation becomes compromised with advancing age in the knockin mice. In middle-aged human *LRRK2*^G2019^ carriers, alterations in serotonin transporter binding have been reported (Wile et al., 2017). Nevertheless, it is important to emphasize that this and other *Lrrk2*^G2019S^ knockin lines of mice do not exhibit age-related dopamine neuron degeneration nor do they exhibit age-related motor deficits mimicking human Parkinson’s (Longo et al., 2014; Yue et al., 2015; Volta et al., 2017; Matikainen-Ankney et al., 2018; Guevara et al., 2020). The mice are thus better models of some non-motor symptoms, exhibiting for example fronto-striatal based cognitive deficits (Hussein et al., 2022) and psychiatric-like behaviors such as those studied here and previously (Matikainen-Ankney et al., 2018; Guevara et al., 2020).

In idiopathic Parkinson’s, symptoms of anxiety and depression are more prevalent in women than in men (Leentjens et al., 2011; Cui et al., 2017). Although the prevalence of anxiety and depression in human *LRRK2* mutation carriers is similar to that in idiopathic Parkinson’s (Gaig et al., 2014; San Luciano et al., 2017; Chen et al., 2020; DeBroff et al., 2023), it is less clear whether there are sex differences in psychiatric symptoms among manifesting *LRRK2*^G2019S^ carriers. One study suggests that depression severity may be similar in male and female mutation carriers (San Luciano et al., 2017), which aligns with the equal penetrance of the mutation in male and female carriers (Marder et al., 2015). Our data in the young adult *Lrrk2*^G2019S^ mice suggest that behavioral stress impacts anxiety-like behaviors in females more significantly than in males, bearing some similarity to idiopathic Parkinson’s. Elevated LRRK2 kinase activity, in the absence of genetic mutation, is found in sporadic Parkinson’s (Kluss et al., 2019; Rocha et al., 2022). As such, behavioral outcomes and regional cell activation analyses of stress effects in *Lrrk2*^G2019S^ mice may have broad relevance to Parkinson’s generally.

Despite broadly similar anxiety-like behavioral responses to 28-*VS* between *Lrrk2*^G2019S^ and wildtype mice, the densities of cFos-labeled cells across brain regions activated under such conditions showed both genotype-related and sex-specific differences. In stressed male and female *Lrrk2*^G2019S^ mice, densities of cFos-labeled cells were affected in mostly similar ways compared to stressed *Lrrk2*^WT^ mice—lower (mPFC, dStr and BLA), slightly higher (dHPC) or unchanged (vHPC). The exception to this pattern was in the NAc. In this structure, cFos densities in male *Lrrk*^G2019S^ mice were significantly higher than all other groups, whereas in female *Lrrk*^G2019S^ mice, cFos density was similar to male and female *Lrrk2*^WT^ mice. It is possible that in male *Lrrk*^G2019S^ mice, the unique combination of dysregulated top-down control of NAc circuitry by mPFC (Britt et al., 2012) and/or the BLA (Stuber et al., 2011) suggested by lowered cFos densities in those structures, coupled with heightened activation of NAc neurons suggested by the elevated density of cFos-labeled cells, reflects abnormal network activity that ultimately increases motivation to feed and/or blunts anxiety associated with the novel arena. Previous studies of wildtype mice have shown that NSF produces elevated densities of cFos-labeled cells in NAc (in comparison with controls exploring an open-field arena), while optogenetically activating an anterior paraventricular thalamus-to-NAc projection during the NSF test increases motivation to consume food in a novel environment and increases time spent in the open arms of an elevated plus maze (Cheng et al., 2018), commonly interpreted as lowered anxiety. Additionally, other forms of chronic stress in wildtype mice increase glutamate release from mPFC terminals onto a subpopulation of BLA neurons that is correlated with heightened anxiety-like behaviors. Decreasing such stress-induced glutamate release reduces anxiety-like behaviors (Liu et al., 2020). Future studies could test the significance of the sex- and genotype-specific cFos labeling patterns described here by interrogating circuit-based contributions to behavioral outcomes.

One limitation in the design of the cFos labeling study is that while 28d-*VS* animals were sacrificed following a final restraint-stress session, there was no comparative group receiving only restraint-stress without prior 28d-*VS*, leaving some uncertainty as to whether differential cFos labeling patterns between *Lrrk2*^G2019S^ and *Lrrk2*^WT^ mice were driven by the cumulative effect of chronic stress, or were driven entirely by an acute episode of restraint stress. Previous studies of wildtype mice and rats have shown that a single episode of acute restraint stress generally increases cFos labeling density in many brain regions (Hsu et al., 2007; Kovács et al., 2018; Kim and Chung, 2021; Han et al., 2023). However, it should be emphasized that the goal here was to compare between genotypes how stressful experience shapes cFos labeling patterns, with less emphasis on exactly what the stressful experience was in either type or duration. Thus, the conclusion stands that a stressful experience drives cFos labeling patterns that differ between sex and genotype. Future studies could address how single episodes of any of the three stressors used here differentially and acutely impact cFos-labeling patterns between genotype.

The mechanistic basis for genotype- and sex-specific differences in anxiety-induced behavioral measures and cellular activation patterns remain speculative. Previous studies of *Lrrk2*^WT^ and *Lrrk2*^G2019S^ male mice subjected to 1-day social defeat stress show significant genotype-specific differences in synaptic properties and intrinsic excitability (Guevara et al., 2020), suggesting that the *Lrrk2*^G2019S^ mutation drives different cellular and synaptic adaptations to other types or durations of behavioral stress. While we did not examine cellular or synaptic properties electrophysiologically or neurochemically in the present study, a previous study of wildtype mice exposed to sub-chronic (6d) variable stress using the same stressors showed significant changes in molecular markers of excitatory synapses in the NAc of female, but not male mice (Brancato et al., 2017). Thus, it is likely that 28d-*VS* drives both genotype- and sex-specific differences in synaptic and non-synaptic adaptations in several, if not all of the stress-relevant brain regions.

Finally, *Lrrk2*^G2019S^ may be differentially influencing stress-relevant brain circuits through exogenous inflammatory mediators. Cells of the peripheral innate immune system express high levels of LRRK2 (Ahmadi Rastegar and Dzamko, 2020), many of which gain access to the brain following periods of behavioral stress, where they play significant roles in modulating cellular and synaptic function in stress-relevant brain regions (Hodes et al., 2015; Weber et al., 2017).

## Data Availability

The original contributions presented in the study are included in the article/supplementary material, further inquiries can be directed to the corresponding authors.
